# Ghana randomized air pollution and health study (GRAPHS): study protocol for a randomized controlled trial

**DOI:** 10.1186/s13063-015-0930-8

**Published:** 2015-09-22

**Authors:** Darby W. Jack, Kwaku Poku Asante, Blair J. Wylie, Steve N. Chillrud, Robin M. Whyatt, Kenneth A. Ae-Ngibise, Ashlinn K. Quinn, Abena Konadu Yawson, Ellen Abrafi Boamah, Oscar Agyei, Mohammed Mujtaba, Seyram Kaali, Patrick Kinney, Seth Owusu-Agyei

**Affiliations:** Mailman School of Public Health, Columbia University, New York, NY USA; Kintampo Health Research Centre, Ghana Health Service, Brong Ahafo Region, Kintampo, Ghana; Lamont-Doherty Earth Observatory of Columbia University, New York, NY USA; Division of Maternal-Fetal Medicine, Vincent Department of Obstetrics and Gynecology, Massachusetts General Hospital and Harvard Medical School, Boston, MA USA

**Keywords:** Household air pollution, LPG, Cookstove, Ghana, Pneumonia, Birth weight

## Abstract

**Background:**

Household air pollution exposure is a major health risk, but validated interventions remain elusive.

**Methods/Design:**

The Ghana Randomized Air Pollution and Health Study (GRAPHS) is a cluster-randomized trial that evaluates the efficacy of clean fuels (liquefied petroleum gas, or LPG) and efficient biomass cookstoves in the Brong-Ahafo region of central Ghana. We recruit pregnant women into LPG, efficient cookstove, and control arms and track birth weight and physician-assessed severe pneumonia incidence in the first year of life. A woman is eligible to participate if she is in the first or second trimester of pregnancy and carrying a live singleton fetus, if she is the primary cook, and if she does not smoke. We hypothesize that babies born to intervention mothers will weigh more and will have fewer cases of physician-assessed severe pneumonia in the first year of life. Additionally, an extensive personal air pollution exposure monitoring effort opens the way for exposure-response analyses, which we will present alongside intention-to-treat analyses. Major funding was provided by the National Institute of Environmental Health Sciences, The Thrasher Research Fund, and the Global Alliance for Clean Cookstoves.

**Discussion:**

Household air pollution exposure is a major health risk that requires well-tested interventions. GRAPHS will provide important new evidence on the efficacy of both efficient biomass cookstoves and LPG, and will thus help inform health and energy policies in developing countries.

**Trial registration:**

The trial was registered with clinicaltrials.gov on 13 April 2011 with the identifier NCT01335490.

## Background

In recent years, global health advocates have come to see household air pollution (HAP) – exposure to air pollution in the home, primarily from cooking with solid fuels – as a prime target for interventions. According to one recent authoritative estimate, HAP accounts for 3.5 million avoidable deaths per year globally, and ranks fourth by disability-adjusted life years (DALYs) among modifiable risk factors [1]. Reliable evidence on the effectiveness of interventions is, however, sparse. Cleaner interventions cost more, and public health resources are scarce. In short, intervention planners need to know: *how clean is clean enough*? And, *what household energy technologies will get us there?*

The burden of disease from HAP arises when incomplete combustion of solid fuels (primarily wood, animal dung, and crop residue) in inefficient cookstoves emits substantial quantities of fine particles (specifically, particles less than 2.5 microns in diameter, or PM_2.5_), carbon monoxide (CO), and a host of other health-damaging pollutants [2]. Combustion may occur indoors, exposing all household members to high levels of air pollution, or it may occur outdoors, mostly exposing cooks and others who are near the fire. Regardless of the hearth location, average exposures are high [3, 4].

An extensive epidemiological literature links these exposures to adverse health outcomes. Systematic reviews and meta-analyses for child acute lower respiratory infections (ALRI) [5], chronic obstructive pulmonary disease [6, 7], lung cancer [6, 8], reduced birth weight [9], and cataract [6] all show evidence of an association with HAP. For cardiovascular disease, evidence for increased risk from biomass smoke exposure comes both from numerous observational studies [10] and from interpolating risks from tobacco smoke and ambient air pollution [11, 12]. Direct empirical evidence of causality is limited, however. Most studies have been observational and, therefore, vulnerable to uncontrolled confounding. In particular, solid fuel use correlates with poverty, and poverty is both difficult to measure and itself a driver of disease through myriad causal channels. Even the rare epidemiological studies that include plausible measures of poverty are prone to endogeneity that can arise from optimizing behavior by household decision-makers.

Few randomized trials linking cookstove interventions to health outcomes have been reported to date. In a pioneering study in Guatemala, Smith et al. reported significant associations between an intervention cookstove and severe pneumonia [13]. One other unpublished trial from India found that rudimentary mud stoves did not deliver expected sustained exposure reduction or health benefits [14]. As of late 2014, four additional trials were currently underway [15], but results were not yet available.

A number of studies have evaluated the effect of clean cookstove interventions on pollution concentrations or exposures [4]. While most have found statistically significant reductions in pollution, none have reported post-intervention concentrations that meet or exceed World Health Organization (WHO) air quality guidelines for PM_2.5_. Three factors push up post-intervention concentrations. Intervention cookstoves may not be particularly efficient, households may continue to use traditional cookstoves alongside the intervention stove, and emissions from sources outside the home may attenuate the benefits of the intervention.

The goal of the present paper is to describe the study design of the Ghana Randomized Air Pollution and Health Study (GRAPHS), a new study designed to provide experimental evidence both on the health effects of HAP and on the effectiveness of specific, policy-relevant clean cookstove interventions. GRAPHS is a cluster-randomized trial that tests the efficacy of both relatively low-cost efficient biomass cookstoves and liquefied petroleum gas (LPG). We deliver these interventions to pregnant women before their third trimester. Our study outcomes are birth weight and physician-assessed severe pneumonia incidence within the first year of life. Demonstration of these benefits could result in a governmental policy to include improved stoves as part of the standard prenatal care package for women of childbearing age in developing countries.

## Methods/Design

We hypothesize that

1. Use of clean cookstoves introduced before the third trimester of pregnancy will lead to a significant increase in average birth weight.
2. Use of clean cookstoves starting before the third trimester of pregnancy will lead to a significant reduction in the risk of physician-assessed severe pneumonia within the first year of life.

We are testing these hypotheses in a cluster-randomized intervention trial involving 1225 maternal-infant pairs in the Kintampo North and South districts of Ghana, West Africa. Thirty-five rural communities were randomly assigned to one of two intervention arms, or to a control arm. Community-based fieldworkers identify pregnant women and enroll them into the study. Gestational age is determined via ultrasound (SonoSite Inc., Bothel, WA, USA) at the enrollment clinic visit [16].

In intervention communities, we distribute intervention cookstoves to pregnant women after enrollment, along with health insurance and an insecticide-treated bed net. The control group receives insurance and an insecticide-treated bed net at enrollment, and an efficient biomass cookstove at the completion of the postnatal follow-up period in the matched intervention communities. We assess real-time personal exposure to CO among all studied participants during pregnancy (four times each) and among the infants during the first year of life (three times each). We monitor subsets of mothers during pregnancy (once) and of the infants (once) for real-time and PM_2.5_ during one of the CO monitoring sessions.

We measure birth weight to the nearest gram using a Tanita BD 585 digital baby scale (Tokyo, Japan). We record the time elapsed between delivery and birth weight measurement, and only birth weights collected within 24 hours of delivery are considered valid. Birth length and head circumference are also measured, and a placental sample is collected for malaria assessment and archiving. Birth measures are obtained for both health facility and home births.

Fieldworkers check for respiratory symptoms at weekly intervals in the home for the first year of the child’s life. They assess symptomatic children for pneumonia using the Integrated Management of Childhood Illness guidelines [17]. Regardless of fieldworker assessment, the study refers all children with symptoms of respiratory infection or who are otherwise unwell to Kintampo Municipal Hospital for clinical evaluation by a study physician. The study provides transportation and assists with follow-up care as needed. The primary case definition for the outcome of severe pneumonia is physician-assessed history of cough or difficulty in breathing and tachypnea and lower chest in-drawing. Table 1 gives our full set of pneumonia outcomes.

**Table 1:** Definitions of pneumonia outcomes

Classification	Signs and symptoms
Primary definition of physician-assessed severe pneumonia	History of cough or difficulty in breathing and tachypnea (fast breathing ≥ 60 breaths/minute in a child aged < 2 months
	≥50 breaths/minute in a child aged 2–11 months) and lower chest in-drawing
Secondary definition of physician- assessed severe Pneumonia 1	History of cough or difficulty in breathing and tachypnea (fast breathing ≥ 60 breaths/minute in a child aged < 2 months
	≥50 breaths/minute in a child aged 2–11 months) and lower chest in-drawing and chest X-ray consolidation or pleural effusion or other infiltrates as defined in a chest x-ray taken within 72 hours of admission
Secondary definition of physician- assessed severe Pneumonia 2	History of cough or difficulty in breathing and tachypnea (fast breathing ≥ 60 breaths/minute in a child aged < 2 months
	≥50 breaths/minute in a child aged 2–11 months) and lower chest in-drawing and an oxygen saturation less than 90 %
Tertiary definition of severe pneumonia assessed by community based fieldworkers	History of cough or difficulty in breathing and tachypnea (fast breathing ≥ 60 breaths/minute in a child aged < 2 months
	≥50 breaths/minute in a child aged 2–11 months)
Primary definition of pneumonia (physician-assessed)	History of cough or difficulty in breathing and tachypnea (fast breathing ≥ 60 breaths/minute in a child aged < 2 months
	≥50 breaths/minute in a child aged 2–11 months) and lower chest in-drawing
Secondary definition of Pneumonia 1	Primary definition and chest X-ray consolidation or pleural effusion or other infiltrates as defined in a chest x-ray taken within 72 hours of admission
Secondary definition of Pneumonia 2	Primary definition and an oxygen saturation less than 90 %

### Study setting

The research builds on an established collaboration between Columbia University in the City of New York and the Kintampo Health Research Center (KHRC) in Ghana. KHRC provides substantial surveillance and research infrastructure to support GRAPHS [18, 19]. In particular, GRAPHS draws heavily on the Kintampo Health and Demographic Surveillance System (KHDSS), which provides the opportunity to identify pregnant women and follow them up using the KHDSS address system [19].

The study area comprises rural communities in the forest-savannah transition zone in the middle part of Ghana. Households in the study region typically cook on traditional three- stone fires. Wood is by far the dominant fuel, though some households use charcoal as a secondary fuel [20]. Outdoor cooking is prevalent during the dry season, but nearly all households also have enclosed or covered kitchen areas for wet season cooking. In pilot studies leading up to the current work, we found 24 h average PM_2.5_ exposure to be approximately 130 μg/m^3^ [20]. Per the KHDSS database, prevalence of smoking is about 10 % in all households in the study area. Cigarette smoking is very rare among women, as most smokers are men. We assess cigarette use among study participants and other household members via questionnaire.

A cluster was defined as a community or group of communities that:

- Is located in Kintampo North or South Districts (the core study area for KHRC)
- Is primarily rural (in practice, this excluded the 2 district capital communities that are made up of approximately 40,000 people);

A woman is eligible to participate in the study if she:

- Is in the first or second trimester of pregnancy (gestational age ≤ 24 weeks; this is to ensure that the intervention is actually delivered and in use by 28 weeks)
- Is carrying a live singleton fetus (twins and higher order multiples are excluded)
- Is the primary cook in her household; and
- Is a non-smoker.

### Interventions

**Fig. 1:**
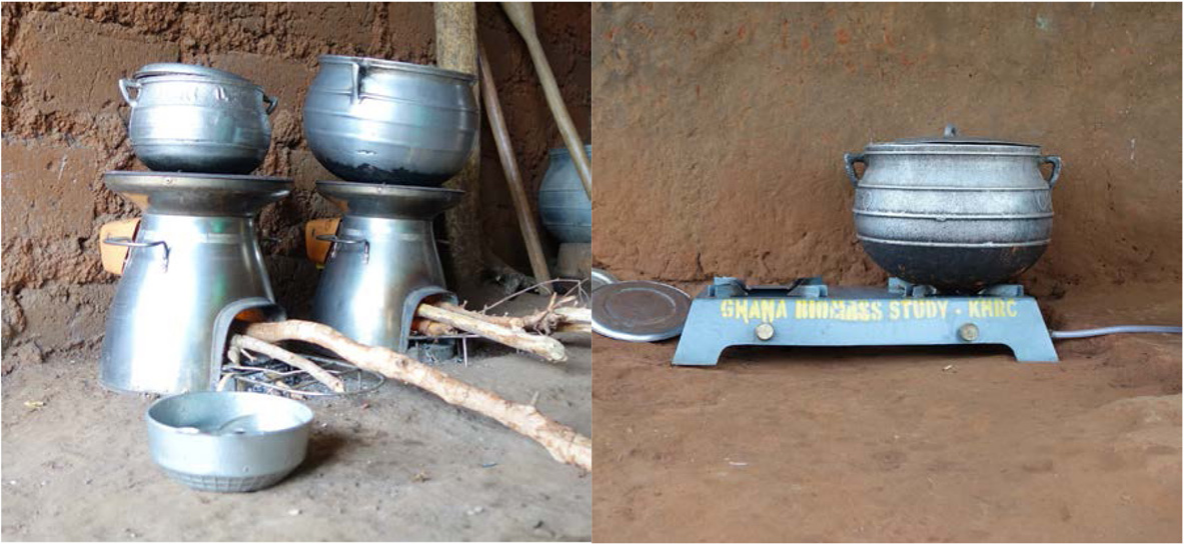
BioLite (left) and liquified petroleum gas (LPG) (right) cookstoves

**Fig. 2:**
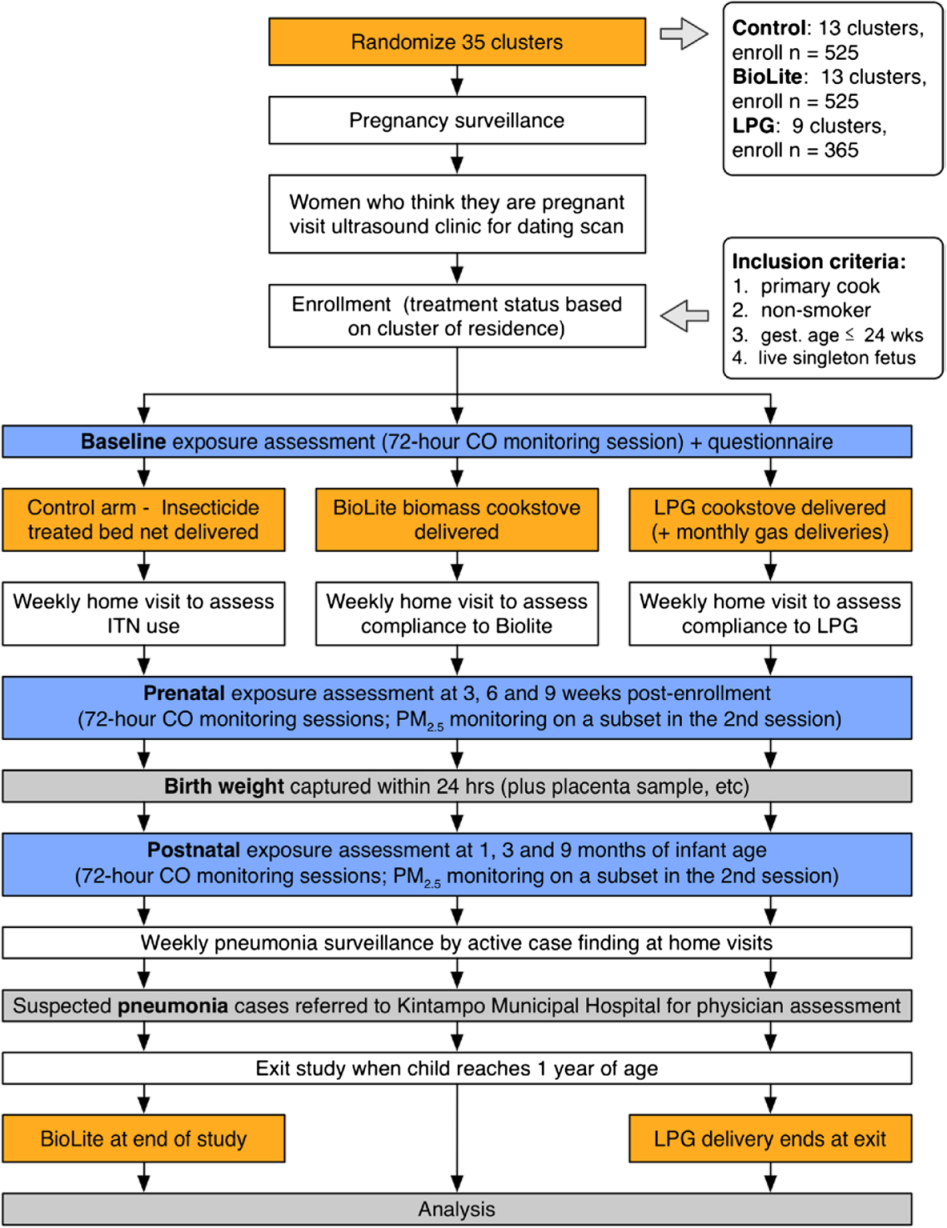
Flow chart

The trial includes a control arm and two intervention arms: an efficient biomass cookstove arm and a LPG arm. Fig. 1 depicts the intervention cookstoves, and Fig. 2 provides an overview of study procedures by arm.

In the efficient biomass cookstove arm – hereafter called the “BioLite arm” – households receive two BioLite HomeStove cookstoves (BioLite Inc., Brooklyn, NY, USA). These stoves burn freely available biomass fuels, but do so in a constrained L-shaped combustion chamber that increases heat transfer efficiency relative to traditional three-stone fires (more energy to the pot per unit of wood). In addition, a fan blows air into the combustion chamber, which improves the combustion efficiency of the system. A thermoelectric generator powers the fan, enabling use without a connection to an electrical grid. While some versions of the BioLite provide power via a Universal S Bus (USB) port, we opted for a version that optimizes combustion efficiency at the expense of USB power.

Study participants in the LPG arm receive a two-burner LPG stove manufactured in Ghana by the Ghana Cylinder Manufacturing Co. (Accra, Ghana). We chose that particular stove because it is readily available in Ghana, both for the current study and for potential future distribution. Enrolled households also receive timely deliveries of LPG gas at no cost to the participants. GRAPHS leased a 5000-kg LPG tank to ensure continuous supply. If households run low on LPG prior to a scheduled delivery, they can contact their fieldworker and the study will make a special delivery. Control arm participants use their own three-stone traditional stoves for the duration of the trial and will receive one BioLite stove at the end of the study period. All participants receive an insecticide-treated net (ITN).

Community-based fieldworkers visit households every week to check up on the intervention stoves and address any problems. In the control areas the community-based fieldworker visits take place as in the intervention arms, and are framed as checkups on the ITNs.

### Power calculations

We performed power calculations based on a model for two-level cluster randomized trials. All calculations were performed using Optimal Design software (OD; [21]), and assume a binary treatment variable. We do not adjust for multiple comparisons because we conceptualize each comparison as testing a distinct hypothesis.

For the birth weight outcome, we calculated the intraclass correlation coefficient to be 0.07 from KHRC birth weight data [22], and used an effect size of 0.32 for the BioLite arm and 0.40 for the LPG arm (derived from [9, 23]). Based on these inputs we calculate a power of 0.84 for the LPG arm and 0.82 for the BioLite arm (with alpha = 0.05) for *n =* 455 for the BioLite and control arms and *n =* 315 for the LPG arm.

For the pneumonia outcome, our power calculations were hindered by the fact that we lacked prior data on acute lower respiratory illness (ALRI) incidence in Ghana. We used data generated by Enwere and colleagues in the Gambia, as part of a vaccine trial [24]. One advantage of the Gambia data is that it includes estimates for a pneumococcal conjugate vaccinated population, as exists in our study area. Physicians carried out all assessments in clinical settings, as will be the case in our study. We also draw on the exposure-response curve relating average PM_2.5_ exposures to relative risks (RR) for ALRI that was developed as part of the 2012 global burden of disease/comparative risk assessment [1, 12].

We define *P*(ALRI) as the probability that a child develop at least one episode of physician-assessed severe pneumonia during the year of surveillance. Multiple occurrences will be analyzed separately. The test statistic, which approximately follows the non-central *t* distribution, was calculated from the parameters in Table 2. The expression for the statistic is complex and is omitted here in the interest of brevity; see [21] for a full presentation (page 141). Using the inputs listed, we calculate that the power of the LPG arm to be 0.98 and the power of the BioLite arm to be 0.89 with an alpha of 0.05.

**Table 2:** Inputs used for acute lower respiratory tract infection (ALRI) power calculation

Parameter	Value	Source	Comment
*P*(ALRI) for controls – all clinical ALRI	0.30	Enwere 2007 (Table 2, column 6, rows 1 and 2)	No ALRI incidence data is available for Ghana; the Enwere data, from Gambia, is the best available analogue. Per the CRA ER, the RR of this exposure level is 2.43
*P*(ALRI) for BioLite intervention arm	0.20	CRA ER curve	Assuming the CRA ER and an exposure reduction of 60 % (130 μg/m^3^ → 78 μg/m^3^), which gives an RR of 1.65
*P*(ALRI) for LPG intervention arm	0.15	CRA ER curve	Assuming the CRA ER and a residual exposure of 20 μg/m^3^, which gives an RR of 1.22
95 % plausible interval for *P*(ALRI) for controls	0.26 – 0.35	Enwere 2007	Estimated from Enwere et al. 2007
Level (α)	0.05	n/a	n/a

*CRA ER* comparative risk assessment exposure response, *LPG* liquefied petroleum gas, *P(ALRI)* probability of a child developing at least one episode of physician-assessed pneumonia during surveillance year, *RR* relative risk

Based on prior research projects undertaken by KHRC, we anticipate an attrition rate of approximately 15 %. Thus, to achieve our goal of *n =* 1225 study participants, we will enroll *n =* 1415. Frequent interactions and strong community support have kept attrition to a minimum to date.

### Randomization

An independent epidemiologist performed final randomization of the clusters, and allocation to control and intervention arms was revealed only after all study personnel were recruited and assigned to their respective clusters, and after initial community sensitization had occurred. Randomization codes will be broken only once the birth weight outcome data collection has finished.

### Exposure assessment

Our exposure assessment strategy is based on the mixture of our pilot experiences in Ghana, which indicated that area sampling was not predictive of personal exposures, the available funds, and the publication record at the time of writing the proposal, which indicated that personal CO can be a good predictor of personal PM_2.5_ exposure [25–27] and that at least 48 hours of sampling was necessary [28, 29]. Furthermore, McCracken et al. [28] have shown that the predictive power of CO for personal PM_2.5_ can be greatly enhanced by taking into account questionnaire data that gather data on other predictors of exposure. As such, our exposure design focuses only on collection of personal samples and questionnaire data.

We use small, lightweight Lascar CO monitors for personal sampling of all women and infants in the proposed study (Lascar Electronics, London, UK). Monitoring sessions last 72 hours each, and occur 7 times (at baseline prior to intervention, 3 additional times during pregnancy, and 3 times after the birth of the baby). The exposure team checks CO monitors using certified span gas every 6 weeks, and instrument-specific readings are adjusted to reflect any drift from accuracy. In addition, we carry out co-located PM_2.5_ sampling in a subset of approximately half of study mothers (1 pre-natal and 1 infant monitoring sessions) to calibrate CO against PM_2.5_. These sampling sessions are also 72 hours long and use the RTI microPEM (RTI International, Research Triangle Park, NC, USA), which provides both gravimetric and nephelometer measurements.

### Data collection and management

We collect questionnaire data via paper forms that are completed in the field by trained field staff. Questionnaires serve to collect data on covariates to include in final analyses and also to track study procedures. Supervisors collect forms from the community-based fieldworkers every week and manually check for completeness and consistency. All data entry is done in KHRC’s computer center, and is completed in duplicate. The trial management team resolves all queries.

Other data are gathered in the clinic. At enrollment, trained midwives perform an ultrasound scan in a clinic setting and submit images to the study obstetrician for quality review and gestational age assignment blinded to trial arm. The exposure team downloads real-time exposure data from the CO and PM_2.5_ devices in KHRC’s exposure lab, and exposed pre-weighed filters are shipped to Columbia University for post-weighing. Real-time exposure data are inspected for quality at time of download. We collect urine from study mothers concurrent with all exposure assessments, and ship aliquoted samples to the US Centers for Disease Control and Prevention for analysis of polycyclic aromatic hydrocarbons (PAHs), with the hope of eventually validating a biomarker of cook smoke exposure.

### Analysis plan

We plan to evaluate the impact of the interventions on birth weight and pneumonia using two methods. First, we will carry out intention-to-treat analyses for both birth weight (assessed as a continuous variable: grams at birth) and incidence of physician-assessed severe pneumonia (assessed as new cases per weeks of child observation). Second, we will carry out exposure-response analysis in which we pool data across arms and model how our outcome variables are related to exposure levels. We will use non-linear models that account for confounders and for clustering. Missing data will be imputed using standard multiple imputation techniques [30]. Both analyses will be carried out with and without adjustments for covariates. In particular, we will control for mother, household, and community characteristics. In the case of birth weight, we will also control for placental malaria.

In addition to these main analyses, we plan several subgroup analyses on our primary outcomes, including comparison of groups defined on residence type (compound versus freestanding households); proximity to main road; gestational age at recruitment; and density of other exposure sources. We also plan to carry out secondary analyses on other birth and health outcomes that we predict will respond to reduction in air pollution exposure.

### Trial monitoring

GRAPHS has an external advisory committee with five members who were chosen for their expertise and for their ability to help disseminate study findings.

A Data and Safety Monitoring Board (DSMB) with four members with expertise in household air pollution, in statistics, and in trial management has been convened to advise regarding any safety issues and review final results. There are no interim analyses planned. Both committees meet periodically to review trial developments. Since the trial does not involve any drugs or medical procedures, the DSMB is not carrying out any interim analyses.

### Consent and ethical approval

We obtain informed consent from each participant prior to enrollment. The study has received unconditional approval by the Institutional Review Board at Columbia University Medical Center, the Ghana Health Service Ethical Review Committee, the Kintampo Health Research Centre Institutional Ethics Committee, and the Partners Human Research Committee. The study is registered with clinicaltrials.gov (NCT01335490).

## Discussion

Household air pollution exposure is a major health risk that requires well-tested interventions. GRAPHS evaluates the efficacy of both clean fuels (LPG) and efficient biomass cookstoves vis-à-vis birth weight and physician-assessed severe pneumonia incidence in the first year of life. The study is set up to provide relevant health outcome data for energy policies in developing countries.

GRAPHS is particularly policy-relevant in Ghana, as it comes at a time when the Ghana government has increased efforts to promote both clean biomass cookstoves and LPG. Spurred in part by the start of domestic LPG production from offshore oil fields, Ghana has recently made a major, public commitment to reduce dependence on uncontrolled combustion for cooking. These commitments appear to be leading to concrete steps to promote LPG access throughout the country and to develop supply chains and subsidies for efficient biomass cookstoves in rural areas. These activities are occurring against a backdrop of a very high burden of disease from HAP. Indeed, the recent Global Burden of Disease/Comparative Risk Assessment process concluded that household air pollution is the most impactful of the modifiable risk factors evaluated for Ghana [31].

We are hopeful that our research will inform these efforts, and similar efforts that are currently underway in other countries, in three ways. First, GRAPHS will provide concrete evidence on the health benefits both from efficient cookstoves and from LPG. These data will provide valuable inputs for benefit-cost analysis of proposed rural energy policies that promote clean cooking. In particular, the direct comparison of LPG to efficient cookstoves will help policy-makers evaluate whether the health benefits of LPG justify its higher cost. Second, our study will help clarify the feasibility and health ramifications of interventions that target pregnant women. Ghana, like most developing countries, has systems in place to reach pregnant women with specific interventions. Third, GRAPHS has built capacity in Ghana for future evaluations of the health implications of energy policies.

### Selecting intervention cookstoves

A central challenge in designing household energy intervention studies is the choice of intervention. To deliver health benefits, interventions must be clean – though as noted above we do not yet know how clean is clean enough – and acceptable to users. Furthermore, if interventions are to be relevant to public health interventions beyond the research program, they must be accessible to the poor. This means that they must be either very inexpensive, or they must be aligned with broader public policy goals and, therefore, plausible targets for subsidies and other government programs.

Our original proposal to National Institute of Health for funding called for the use of locally available rocket stoves. This approach grew out of our unpublished pilot work that showed that such stoves could reduce exposures by approximately 50 %; similar estimates for Ghana have been published by another group [32]. After receiving the grant and convening our external advisory committee, we came to believe that this exposure reduction was unlikely to result in detectible changes in health outcomes. In particular, preliminary exposure response data from the RESPIRE trial [13] suggested that a 50 % exposure reduction would require a sample size approximately twice that which we were funded to study.

This observation prompted a long search for an alternative intervention. We piloted industrially produced rocket stoves, but found that they performed no better than the made-in-Ghana variant. We evaluated the Philips Cookstove (Royal Philips, Amsterdam, The Netherlands), which can be very clean thanks to forced-draft combustion [33], but ultimately rejected it because it requires an external power source to charge its battery and also because its top-loading design seemed incompatible with Ghanaian cooking practices. The BioLite HomeStove appeared to address both of these issues – its thermoelectric system provides electricity for forced draft combustion without a grid connection, and its stable, side-loading design emulates the rocket stoves that we had successfully piloted. We initiated discussions with BioLite in 2010, but at that time they were still developing their technology.

We also started investigating an LPG intervention. LPG is very clean [33] and very convenient, but we were uncertain of its public health relevance given its high cost and limited availability in Ghana. In 2012 we learned, however, that Ghana had laid plans for a substantial program to increase access to LPG both in rural and urban areas [34] and that Ghana’s Ministry of Energy had initiated a “Rural LPG Program” that was delivering free cookstoves and subsidized gas to rural communities. This support from the government was sufficient to convince the GRAPHS team that LPG was indeed a germane alternative.

We remained convinced, however, of the need to include an efficient biomass arm. While our interactions with the Government of Ghana had persuaded us that LPG was worth testing, we remained hopeful that efficient biomass combustion would provide a substantially less costly intervention for future large-scale interventions. Between 2010 and 2012 BioLite developed a production model of their HomeStove, and we began pilot testing in spring 2012. Initial units proved unreliable, but after several iterations we were satisfied that the BioLite was robust to long-term use. We launched enrollment in fall 2013 when the first shipment of BioLite stoves reached our study area in Ghana.

Our intervention entails providing participating households with clean cookstoves and, in the case of the LPG intervention, with an adequate supply of fuel. As with many public health interventions, we cannot ensure perfect compliance. While we try to minimize use of the traditional three-stone fires in intervention households through weekly stove use encouragement visits, we have observed that many households continue to use traditional stoves for certain cooking tasks (a practice sometimes referred to as “stove stacking” [35]). We address this in three ways. As discussed below, we are currently undertaking a separate study to objectively quantify stove use across arms and to link it to household characteristics. Additionally, we carry out weekly questionnaire-based assessments of stove use; these data will be available as a covariate in our adjusted analyses of our main health outcomes. Third, we collect extensive personal exposure data, which capture emissions both from continued use of incumbent stoves and from other nearby sources. These data will be used to estimate exposure-response relationships for our main outcomes.

### Exposure assessment challenges and goals

Most previous studies of childhood health and biomass cooking have relied on qualitative assessments of exposure based on cooking fuels, stove types, and/or time spent near fires [36]. To characterize exposure/response relationships, and thus to define evidence-based targets for future stove interventions, quantitative assessment of personal exposures is needed. Personal exposure assessment is preferred since exposures are affected by behavior patterns in relation to the kitchen and cook fire. Ideally, personal sampling would be of sufficient duration to characterize average exposures during pregnancy and the neonatal period. Also, collection of particle samples on filters for later compositional analysis would facilitate examination of pollution species that may be responsible for adverse effects, potentially leading to greater mechanistic understanding. In spite of new developments in technology, personal sampling remains a challenge. The current project optimizes exposure assessment within the constraints of available technology and funds. In particular, large-scale, multi-day personal PM exposure data remains elusive because personal sampling devices remain cumbersome, expensive, and battery-limited.

As described above, our strategy relies heavily on our ability to predict PM_2.5_ from CO. At the time of writing the proposal, strong correlations between concentrations of PM_2.5_ and the more easily measured pollutant CO had been observed in both in personal and kitchen settings [25, 26]. Since then, however, one study has observed a poor (non-significant) correlation between predicted personal PM_2.5_ (i.e. predicted from the kitchen relationship of PM_2.5_ and CO and measured personal CO) and measured personal PM_2.5_ (Dionisio et al. [29]). McCracken et al. [28] have shown that the predictive power of CO for personal PM_2.5_ can be greatly enhanced by taking into account questionnaire data that include other predictors of exposure. Overall, these results support the use of CO as a proxy for PM_2.5_ in situations where a cookstove is the predominant source for both CO and PM_2.5_ [37], and where detailed information is available on behavior during monitoring sessions. The GRAPHS community field-worker administers a 24 h recall questionnaire focused on exposure assessment issues to the mother for each of the 3 days within the 72 h monitoring session.

### Future opportunities

GRAPHS establishes a cohort of considerable interest for long-term follow-up. We have so far launched four additional sub-studies that will open the way for additional hypothesis testing.

The first study tests the effect of cookstove interventions on the prevalence of pneumonia etiologies. It has been developed in collaboration with the Center for Infection and Immunity at Columbia University. We deploy multiplex MassTag polymerase chain reaction (PCR) analysis targeting genetic material from a panel of respiratory viral and bacterial pathogens in nasopharyngeal samples from pneumonia cases and healthy controls from the GRAPHS cohort [38]. Understanding whether and to what extent cookstove interventions differ in their impacts on pneumonia of different etiologies will help greatly in the interpretation of results from household air pollution research, and in predicting the effects of interventions in settings with varying pathogen prevalence. Previous studies have suggested that exposure to household air pollution causes bacterial pneumonia but may not cause viral pneumonia, while others have found no evidence of a differential effect. Analysis is currently underway at the Center for Infection and Immunity at Columbia University, and additional samples are being collected during ongoing surveillance for pneumonia cases.

The second study assess user uptake of intervention cookstoves (R01 ES 024489). Limited past research has shown that the demand for clean cookstoves is low, and that households continue to use traditional hearths even when they have clean stoves [39]. These behaviors threaten to undermine cookstove intervention programs. The adoption study is structured around four main activities. We will start by measuring stove use in our current intervention and test a series of hypotheses about household characteristics and cultural factors that predict increased use of clean stoves. Second, we will assess the effects of stove type and usage on household time allocation, using time activity diaries to test the hypothesis that clean cookstoves free up substantial time for household members, particularly women. Third, we will carry out a randomized trial to test the effects of health education delivered by community health workers and by local opinion leaders. Finally, we will use a simple bidding procedure to elicit willingness-to-pay for high efficiency cookstoves, and will examine how willingness-to-pay varies as a function of the way in which the stove is marketed.

Third, GRAPHS provided a platform for recently completed pilot work in Ghana that established the feasibility of ambulatory blood pressure (ABP) measurements in rural Africa. ABP monitoring entails fitting study participants with a blood pressure cuff and a small device that automatically measures blood pressure periodically. We deploy ABP monitors for 24 hours and record blood pressure every 20 minutes. This is the first time such measurements have been made in rural Africa. The research focuses on ABP obtained over 24 hour because it is the best available short-term, independent marker of elevated long-term cardiovascular risk. In addition, ABP monitors worn alongside personal air pollution exposure monitors can detect acute BP responses to spikes in exposure – ABP thus measures blood pressure in the settings where exposures occur. The clinical cardiovascular disease outcomes that are of ultimate concern have long latencies, but prior work has shown that clinic-assessed blood pressure responds quickly to reductions in household air pollution. ABP monitoring can identify several distinct blood pressure phenotypes that have been associated with worse cardiovascular outcomes and increased mortality.

Fourth, we aspire to follow the children born to the GRAPHS cohort to assess cognitive development, lung development, and respiratory infections to test the hypothesis that all three are harmed by air pollution exposure in utero. We are currently piloting the Bayley Scales of Infant Development [40], with the goal of deploying it to the cohort at age 3.

## Trial status

The trial was actively recruiting study participants at the time of submission.

## Abbreviations

ABP: ambulatory blood pressure
ALRI: acute lower respiratory infections
CO: carbon monoxide
DALYs: disability-adjusted life years
DSMB: Data and Safety Monitoring Board
GRAPHS: Ghana Randomized Air Pollution and Health Study
HAP: household air pollution
KHDSS: Kintampo Health and Demographic Surveillance System
KHRC: Kintampo Health Research Center
ITN: insecticide-treated net
LPG: liquefied petroleum gas
OD: Optimal design software
PAHs: polyaromatic hydrocarbons
PCR: polymerase chain reaction
PM_2.5_: particulate matter less than 2.5 microns in diameter (fine particles)
RR: relative risk
USB: Universal Bus
WHO: World Health Organization
